# Human endogenous retrovirus profiling reveals heterogenous expression in cutaneous melanoma

**DOI:** 10.3389/fonc.2026.1708501

**Published:** 2026-03-26

**Authors:** Tongyi Fei, Bhavya Singh, Nicholas Dopkins, Jez L. Marston, Jasmine H. Francis, Douglas F. Nixon, Alexander N. Shoushtari, Matthew L. Bendall

**Affiliations:** 1 Division of Infectious Diseases, Department of Medicine, Weill Cornell Medicine, New York, NY, United States; 2 Department of Immunology and Immunotherapy, Icahn School of Medicine at Mount Sinai, New York, NY, United States; 3 Northwell Health, New Hyde Park, NY, United States; 4 Feinstein Institutes for Medical Research, Institute of Translational Research, Manhasset, NY, United States; 5 Zucker School of Medicine at Hofstra/Northwell-Hofstra University, Hempstead, NY, United States; 6 Ophthalmic Oncology Service, Memorial Sloan Kettering Cancer Center, New York, NY, United States; 7 Department of Ophthalmology, Weill Cornell Medicine, New York, NY, United States; 8 Melanoma Service, Department of Medicine, Memorial Sloan Kettering Cancer Center, New York, NY, United States; 9 Department of Medicine, Weill Cornell Medicine, New York, NY, United States; 10 Department of Biostatistics and Bioinformatics, Computational Biology Institute, Milken Institute School of Public Health, The George Washington University, Washington, DC, United States

**Keywords:** cutaneous melanoma (CM), human endogenous retrovirus (HERV), immunotherapy responsiveness, metastasis, syncytin-2

## Abstract

**Background:**

Cutaneous melanoma (CM) is a cancer of the pigment-producing melanocytes of the skin. Advances in treatment with immune checkpoint inhibitors (ICI) have greatly improved the outcomes, although not all patients with CM respond to ICI. Emerging evidence shows that ICI responsiveness is correlated with the expression of certain human endogenous retroviruses (HERVs). We aimed to investigate the relationships between HERV expression and outcomes in patients with CM.

**Methods:**

We characterized HERV expression from 330 patients with primary or metastatic CM using Telescope, a computational software package that calculates the expression of HERV loci from bulk RNA sequencing datasets.

**Results:**

We found that HERVs expression differed between primary and metastatic CM. Using HERV expression alone, three subtypes of metastatic CM had distinct survival outcomes. Among the differentially expressed HERV loci in metastatic and treatment non-responders, we identified HERV-FRD which has the potential to produce the endogenous retrovirus group V Env polyprotein (syncytin-2) precursor.

**Conclusion:**

Our analyses demonstrate the unique features of HERV expression in CM metastases and in ICI responsiveness. These exploratory findings identify modulated HERVs in CM, and further studies should be developed to determine whether HERV expression has any predictive or clinical utility.

## 1 Introduction

Melanomas are common malignant tumors that originate from melanocyte cells that produce the pigment melanin. The majority of melanomas are skin melanomas [cutaneous melanomas (cm)]. There are environmental, genetic, and immunological factors that contribute to the onset and severity of CM, such as exposure to ultraviolet (UV) light and the presence of a BRAF mutation (1). The 5-year relative survival rate varies significantly by stage at diagnosis, with a survival rate of 29.8% when metastases have spread beyond the skin or lymph nodes (2). The mortality rate has fallen to 30% over the past decade with advances in immune checkpoint inhibitor (ICI) therapy.

ICI blocks co-inhibitory receptors on T cells which normally act as immunoregulatory checkpoints to allow for better T- cell-mediated recognition of tumor antigens (3). The first successful immunotherapy attempt in metastatic melanoma was performed with an anti-CTLA-4 blocking antibody (4). Soon after, blocking antibodies to PD-1 and PD-L1 were shown to improve CD8+ T cell function, leading to developmental approval (5). Despite their success in the clinic, some patients are nonresponsive to ICI therapies, highlighting the need for alternative strategies and better understanding of why some ICI treatments fail (1).

Human endogenous retroviruses (HERVs) are ancient integrated retroviruses that encompass around 9% of the human genome (6, 7). Some HERVs maintain certain regulatory and coding functions (8), including retroviral gene products, such as the capsid (*Gag*) and retroviral envelope (*Env*) proteins (8). While some HERVs have been co-opted for beneficial processes, sometimes their deregulated activity leads to pathological processes which are detrimental to host health. To restrict HERV-driven pathology, our germline and somatic cells possess a complex array of regulatory networks that control the expression of HERV elements (9). Deregulated HERV expression can drive oncogenesis through disrupting the host regulome, dysregulating tumor suppressor genes, or promoting the expression of established oncogenes (8). Previous studies have related ICI responsiveness to the expression of HERVs (10–12), but how HERV activity directly relates to metastatic potential is unclear. An investigation into the functional roles of HERV expression in ICI suggests that HERVs might drive immune evasion by triggering the expression of PD-L1 in immune cells (13).

The World Health Organization classification of melanomas identifies nine distinct subtypes based on epidemiological, clinical, histopathological, and genomic features, of which four are CM: superficial spreading/low cumulative solar damage (CSD) melanoma, lentigo maligna/high CSD melanoma, acral melanoma, and nodular melanoma (14, 15). CM is further classified into four clusters based on genotype: mutant BRAF, mutant RAS, mutant NF1, and Triple-WT, each with their distinct gene expression, immune infiltration signature, and survival outcome (16). However, genotypic clustering of CMs does not consider the landscape of HERV expression. Studies in other malignancies have shown that the oncogenic derepression of HERV activity can influence immune recognition and the oncogenesis of the local tumor microenvironment (17, 18), provide a source of recurrent tumor specific neoantigens (19–25), and predict prognostic outcomes (10, 25–27).

While HERV expression can be established from RNA sequencing datasets, there are difficulties in quantification, including their low abundance, high sequence homology, and the presence of chimeric transcripts spliced to canonical coding genes (28). To account for these concerns, we applied the bioinformatic pipeline Telescope to measure HERV abundances in the transcriptomes of 330 CM samples retrieved from the Cancer Genome Atlas Program Cutaneous Melanoma Study (TCGA-SKCM) (16) and an additional dataset specifically focused on prognostic outcomes in CM patients receiving anti-PD1 therapy (29).

We initially identified several differentially expressed HERVs in metastatic CM compared to primary CM. We then categorized three clusters based on HERV expression alone and observed a pattern of HERV downregulation in CM defined by poorer disease prognoses. We also identified an upregulated HERV-FRD with an open-reading frame (ORF) putatively encoding the *Env* “syncytin-2”. In this exploratory study, we found that changes in HERV expression were associated with CM metastases, ICI responsiveness, and CM subtypes.

## 2 Methods

### 2.1 Data retrieval

Bulk RNA-Seq fastq files were obtained from publicly available studies. Briefly, CM samples were downloaded from the TCGA-SKCM (16), containing 65 samples from patients with primary tumors and 265 samples from patients with metastatic tumors (**Supplementary Table 1A**). Tumor mutational burden (TMB) information was calculated using maftools 2.16.0 (30) from the somatic mutation counts provided by TCGAbiolinks 2.28.3 (31). We categorized the TMB into four levels according to published literature (32). After conducting differential expression analysis between primary and metastatic subgroups, we subsetted the metadata of all 204 metastatic samples measures for cancer stage, gender (125 male, 82 female), specimen site, biopsy site, TMB information, and patient survival outcome (**Supplementary Table 1B**).

To validate that differentially expressed HERVs captured in the metastatic samples were not driven by the site of specimen, we downloaded bulk RNA-Seq fastq of normal tissues. To determine associations between HERV expression and immunotherapy treatment outcome in CM, we conducted two independent analyses with two separate datasets. The TCGA-SKCM dataset was subsetted into 20 patients having the immunotherapy response recorded, which includes 19 pre-immunotherapy treatment samples and one on- or post- treatment sample (**Supplementary Table 1C**). In addition, we downloaded the dataset containing outcome records using the Response Evaluation Criteria in Solid Tumors (RECIST) categories (29, 33), which included 27 metastatic CM samples (26 pre-immunotherapy treatment and one on-treatment). For both datasets, the outcome category was reclassified as binary variable “non-responder” (stable disease or progressive disease) and “responder” (complete response or partial response) (**Supplementary Table 1D**). Demographics and metadata from all utilized studies were organized by source type (**Supplementary Table 1**). All sequencing data used in the analyses are from publicly available datasets and are human subjects exempt (**Supplementary Figure 1**).

### 2.2 HERV quantification

HERV quantification and analysis were performed according to published protocols using the Telescope pipeline (10, 34) v.1.3.0. Briefly, fastq files were downloaded from dbGap under project numbers phs000178.v11.p8 (16) and GEO under accession number GSE7822018. The samples were then aligned to the human genome build 38 (hg38) reference version (35) using STAR with the following parameters: “--outSAMattributes NH HI NM MD AS XS --outSAMtype BAM Unsorted --quantMode GeneCounts --outFilterMultimapNmax 200 --winAnchorMultimapNmax 200 --outSAMunmapped Within KeepPairs” to quantify gene expression while preserving the multi-mapped reads to yield BAM files for the subsequent quantification of HERVs. Transcriptome assembly and annotation were then performed with Stringtie. We next used the Telescope assign pipeline on the final BAM files with the following parameters: “ --exp_tag inform --theta_prior 200000 --max_iter 1000” to quantify HERV and long interspersed nuclear element (LINE) counts. The Snakemake workflow for alignment and expression quantification is documented on GitHub (https://github.com/nixonlab/TCGA_SKCM_mets for the TCGA dataset (16), https://github.com/nixonlab/Mets_CM_bulk for the anti-PD1 dataset (29)). Count matrices were then loaded into R for downstream differential expression analyses of HERV loci.

Transposable element (TE) transcripts that were non-HERVs were excluded from the analysis to reduce dimensions in the differential expression analysis. Genes and HERVs with fewer than five RNA read counts were filtered out from the downstream workflow. We used DESeq2 1.36.0 for differential expression analysis and standard normalization. For primary and metastatic CM, PCAtools 2.8.0 was used to calculate important variances and visualize the principal component analysis (PCA) space annotated by available metadata. The bottom 30% of the genes and 20% of the HERVs that explained the variances were excluded from downstream analyses. Filtered datasets were analyzed with the following differential expression calculation workflow: a Wald test was performed using DESeq2 (v1.36.0). Taking into account the large cohort size of TCGA, we established a significance threshold of a Bonferroni-adjusted *p*-value < 0.01 and absolute value of log2 fold change > 2 for transcripts to be considered differentially expressed.

### 2.3 Feature selection

Feature selection was applied to identify the most significant HERVs that differentiated metastatic and primary samples. A likelihood ratio test (LRT) for differential expression was performed using DESeq2 (v1.36.0), with the same adjusted *P*-value and log2 fold change threshold as the Wald test. Boruta test was performed using Boruta (v8.0.0) and LASSO test performed using glmnet (v4.1.7) on the variance-stabilizing transformed dataset. The HERVs and genes selected by the feature selection methods were visualized with pheatmap (v1.0.12). We used EnhancedVolcano (v1.14.0) to compare gene and HERV expression between metastatic and primary samples. For gene functional mapping, we performed GO enrichment analysis (36) using clusterProfiler (v4.4.4) (37) and https://bioconductor.org/packages/release/data/annotation/html/org.Hs.eg.db.html (v3.15.0), which maps Entrez gene identifier to NCBI gene accession numbers (38).

### 2.4 Survival analysis

For the prognostic HERV marker analyses, consensus clustering using ConsensusClusterPlus 1.60.0 was performed on the 207 metastatic CM samples with complete metadata. Three clusters based on HERV expression were compared to the clusters published by the original literature, calculated based mostly on gene expression (16). Rand indexes were calculated between clusters and metadata columns, and the expression data were visualized with a PCA using PCAtools 2.8.0 to investigate which metadata factors might contribute to the clustering. Kaplan–Meier (KM) survival analyses were performed comparing the HERV clusters with survival (v3.5.5) and survminer (v0.4.9). The downstream analysis followed a similar feature selection and visualization pipeline as metastatic vs. primary described above, where each cluster was compared to the other clusters. It was not possible to perform a multivariable survival analysis adjusting for known prognostic covariates.

### 2.5 Treatment responsiveness analysis

For the outcome analysis, we analyzed the TCGA data subset and additional RECIST data subset independently since their outcome classification methods differed. The RECIST anti-PD1 dataset contained two batches collected from UCLA and Vanderbilt and thus was first batch-corrected using ComBat_seq in sva (v3.44.0). Then, both analyses followed similar PCA visualization and differential expression analyses as described above with minor modifications. Due to the potential impact of batch effects yielding false negatives, additional statistical stringencies were incorporated by elevating the *p*-value threshold to 0.05 when determining significance in the differential expression of HERVs, and only the Wald test was used. Feature selection was not needed, and the more lenient *p*-value cutoff was established based on the smaller sample size and the exploratory nature of this analysis. Differentially expressed HERVs from the analyses were summarized and compared by selecting the significant HERVs from each analysis (absolute log2 fold change > 2 and *p*-value < 0.01 for metastatic vs. primary and in-metastatic clustering; *p*-value < 0.05 and absolute log2 fold change > 2 for response outcome comparison).

### 2.6 HERV ORF identification

The functionality of HERVs was annotated with the TE meta-annotation provided by Telescope (https://github.com/liniguez/Telescope_MetaAnnotations) (10, 34). Details of the downstream analysis R code can be found on GitHub in downstream R sub-folder (https://github.com/nixonlab/TCGA_SKCM_mets/tree/master/Met_Mel_Downstream_R). To predict possible HERV contributions to the proteome, HERVs were selected by the following criteria: co-upregulated or co-downregulated in at least two of the three analyses (metastatic vs. primary, worst prognostic metastatic cluster vs. all other metastatic, or non-responder vs. responder in either immunotherapy datasets).

The selected HERVs were annotated with the meta-annotation, sequences extracted from the hg38 database (35), and then the ORFs were investigated using NCBI ORFfinder (38) with minimal ORF length of 150 nucleotides, standard gene code, and “ATG only” start codon. For the longest three non-overlapping ORFs with the same strand, if the HERV was on the antisense strand, we only filtered ORFs that were antisense so that the proteins predicted were sense-stranded, and *vice versa*; if the ORFs were overlapping, then only the longer one was used). We searched with BlastP in the UniProtKB/Swiss-Prot (swissprot) database (38) for the best hit of *Homo sapiens* proteins. If hits were found, protein ranges and predicted sequences were recorded and saved for epitope binding potential analyses using NetMHCPan (v4.0) (39) (https://services.healthtech.dtu.dk/services/NetMHCpan-4.0/). All matched proteins were saved in fasta format and uploaded to the web interphase. All peptide lengths were selected (8 – 14mers), and HLA-A*02:01 was used for the human leukocyte antigen (HLA) allele. The threshold for a strong binder was set to be 50% rank and the weak binder set to 2% rank. When recording the binder prediction results, only strong binding and non-overlapping peptides were recorded (if there were over-lapping peptides, the one with the highest score was selected).

### 2.7 Expression of ERVFRD-1 in CM tumors and normal tissues

HERVFRD_6p24.2 overlaps with the canonical gene ERVFRD-1 (ENSG00000244476). In order to compare expression of this locus with a very large dataset of normal tissues, we used read counts for ERVFRD-1 in CM samples (see 2.2) and from GTEx v11 analysis, obtained from https://gtexportal.org/home/downloads/adult-gtex/bulk_tissue_expression. Read counts for 19,788 GTEx samples were normalized by library size to obtain counts-per-million (CPM), which were merged with CM expression estimates. Detailed analysis could be found on https://github.com/nixonlab/tcga-skcm-r2-gtex-analysis.

### 2.8 Statistics

All statistical analyses were performed using R and relevant Bioconductor packages. For differential expression analyses of gene and HERVs, we applied the default Wald test within DESEQ2 to identify the differential expression between groups. For evaluation of metastatic vs. primary and clustering, gene and HERV transcripts were considered significant if they met both a Bonferroni-adjusted (correct for multiple testing) *p*-value threshold of less than 0.01 and an absolute log2 fold change threshold larger than 2. To evaluate between ICI treatment responsiveness groups, due to batch effect and smaller sample sizes, adjusted *p*-value threshold was set to be less than 0.05 while log2 fold change threshold stayed the same. Likelihood ratio, Boruta, and LASSO regression tests were performed on variance-stabilizing transformed data for feature selection. The same significance thresholds for *p*-values (0.05 for responsiveness, 0.01 for others) and log2 fold change absolute values (≥2) were used. For PCA, the bottom 30% of genes and the bottom 20% of HERVs contributing variance were excluded before performing dimensionality reduction. Similarity between calculated clusters and clinical metadata variables were evaluated using adjusted Rand index. GO enrichment analysis was performed with the statistical significance determined by adjusted *p*-values default to the clusterProfiler package. Differences in survival between groups were assessed using log-rank test. Significant differences in mean gene expression (CPM) among groups was tested using the Kruskal-Wallis test, pairwise differences between groups were tested using the Wilcoxon rank sum test, the adjusted *p*-value significance threshold was 1e-3.

## 3 Results

### 3.1 Differential HERV expression between primary and metastatic melanoma

**Figure 1:**
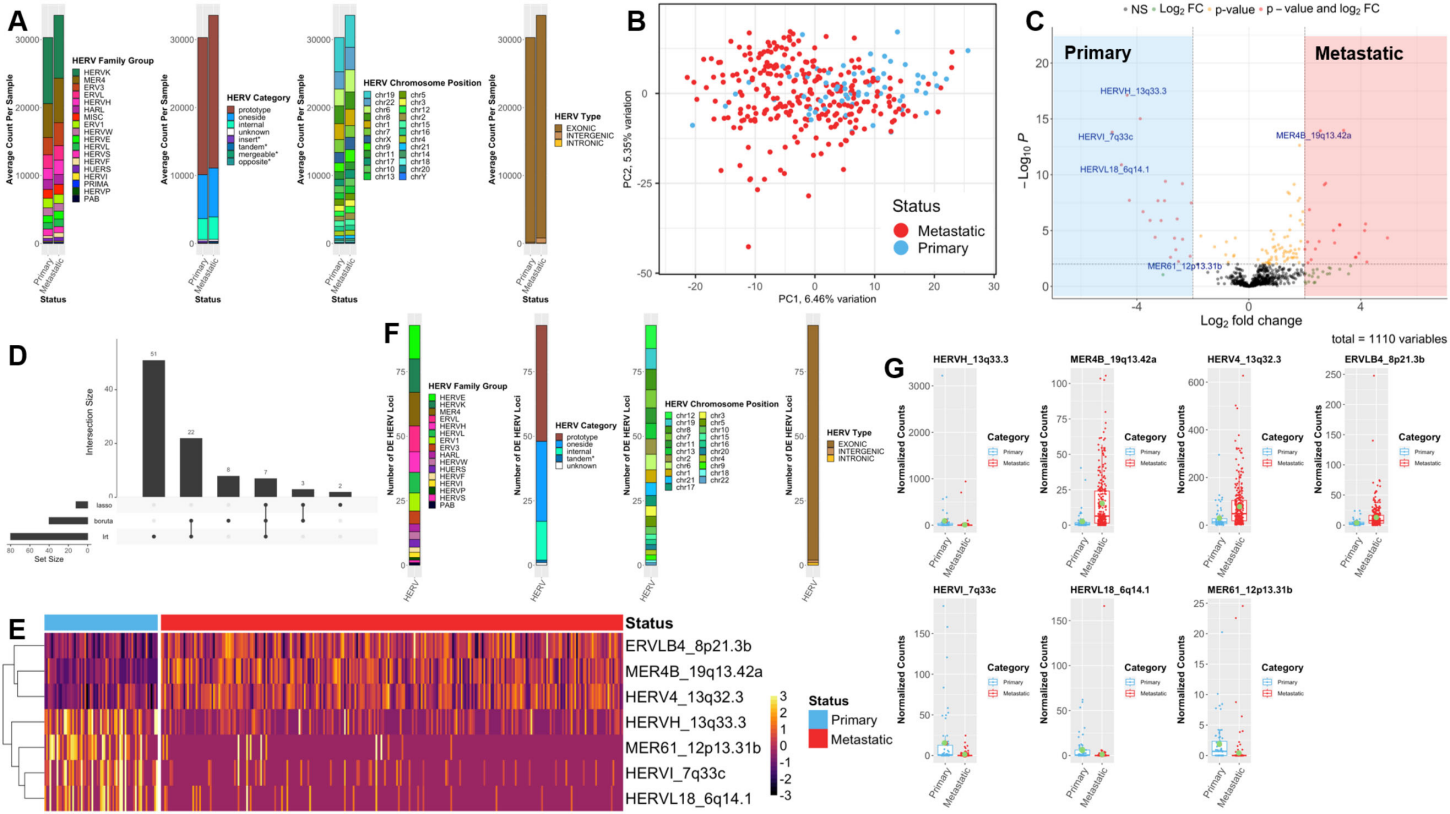
Primary and metastatic melanoma samples have distinct gene and HERV expression profiles and should be analyzed separately. **(A)** Summary stack plots of average counts of HERVs per sample for primary and metastatic melanoma, for different summary categories. Summary categories are HERV family groups, HERV functional category, HERV chromosome position, and HERV gene position type. Categories are ordered by count. **(B)** PCA of HERV expression in all TCGA-SKCM samples. HERVs representing the lowest 20% of variation are removed. Colored based on the sample status (metastatic or primary tumor). **(C)** Volcano plot of differential HERV analysis between primary and metastatic melanoma samples. P values and log fold change are calculated with the Wald model. Marked genes are consensus feature selected genes using LRT, BORUTA and LASSO methods. P-values cutoff = 0.01, and Log2FC cutoff = +/- 2. **(D)** Upset plot showing the number of differential HERVs selected by each feature selection methods. **(E)** Heatmap showing expression of 7 consensus feature selected HERVs in each sample. **(F)** Summary stack plots of all the feature selected differentially expressed HERVs comparing primary and metastatic melanoma. Summary categories are HERV family groups, HERV functional category, HERV chromosome position, and HERV gene position type. Categories are ordered by number of HERV loci. **(G)** Normalized count plots of the consensus feature selected HERVs in primary and metastatic melanoma samples. The line indicates median and green dot indicates mean normalized count of each sub-group.

To test which HERVs were differentially expressed between the metastatic and primary samples, we compared the average HERV transcript count per sample (**Supplementary Figure S1**). In general, metastatic samples had higher HERV RNA counts per sample, but the distribution of the HERV family, group, category, and chromosomal location was similar between the primary and metastatic samples with most of the HERVs being exonic (**Figure 1A**). When plotted on the PCA space, multidimensional scaling identified a trend of separation between status based on HERV expression alone (**Figure 1B**), although certain primary and metastatic samples overlapped. A similar separation trend was observed for gene expression (**Supplementary Figure 1A**). We suspect that the procedure to reduce noise from the RNA sequencing dataset might have removed some variances, causing the mix of some primary and metastatic samples.

We ran DESeq2 comparing the 265 metastatic to 65 primary CM samples. We found that more HERVs were downregulated (defined by smaller *p*-value and larger absolute log 2 fold change) (**Figure 1C**), consistent with the gene expression profile between primary and metastatic CM (**Supplementary Figures 2A-C, F**). Overall, 93 total HERVs were selected by the three feature selection methods (LRT, LASSO, Boruta), and seven HERVs (three upregulated and four downregulated) in the metastatic samples were identified by all three selection methods (**Figures 1D, E**; **Supplementary Figure 2G**).

In the 93 selected DE loci, HERV-K and HERV-E, have been described in other studies to be dysregulated in multiple cancer tumor microenvironments (40–42) (**Figure 1F**). The top selection mostly belonged to the immune regulatory function group, and some belonged to the keratinization and skip development (**Supplementary Figures 2D, E**). By plotting the counts for the seven-consensus selected HERVs, we determined that despite HERV expression showing variability within the sample group, MER4B 19q13.42a, HERV4 13q32.3, and ERVLB4 8p21.3b were significantly upregulated, and HERVI 7q33c and HERVL18 6q14.1 showed clear downregulation in CM metastasis (**Figure 1G**). HERV expression profiles were distinct between primary and metastatic samples, with the caveat that mixing metastatic and primary samples in KM survival analyses might have induced lead-time bias, thus skewing the results (43, 44).

### 3.2 Survival analysis of primary melanoma HERV expression sub-clusters

**Figure 2:**
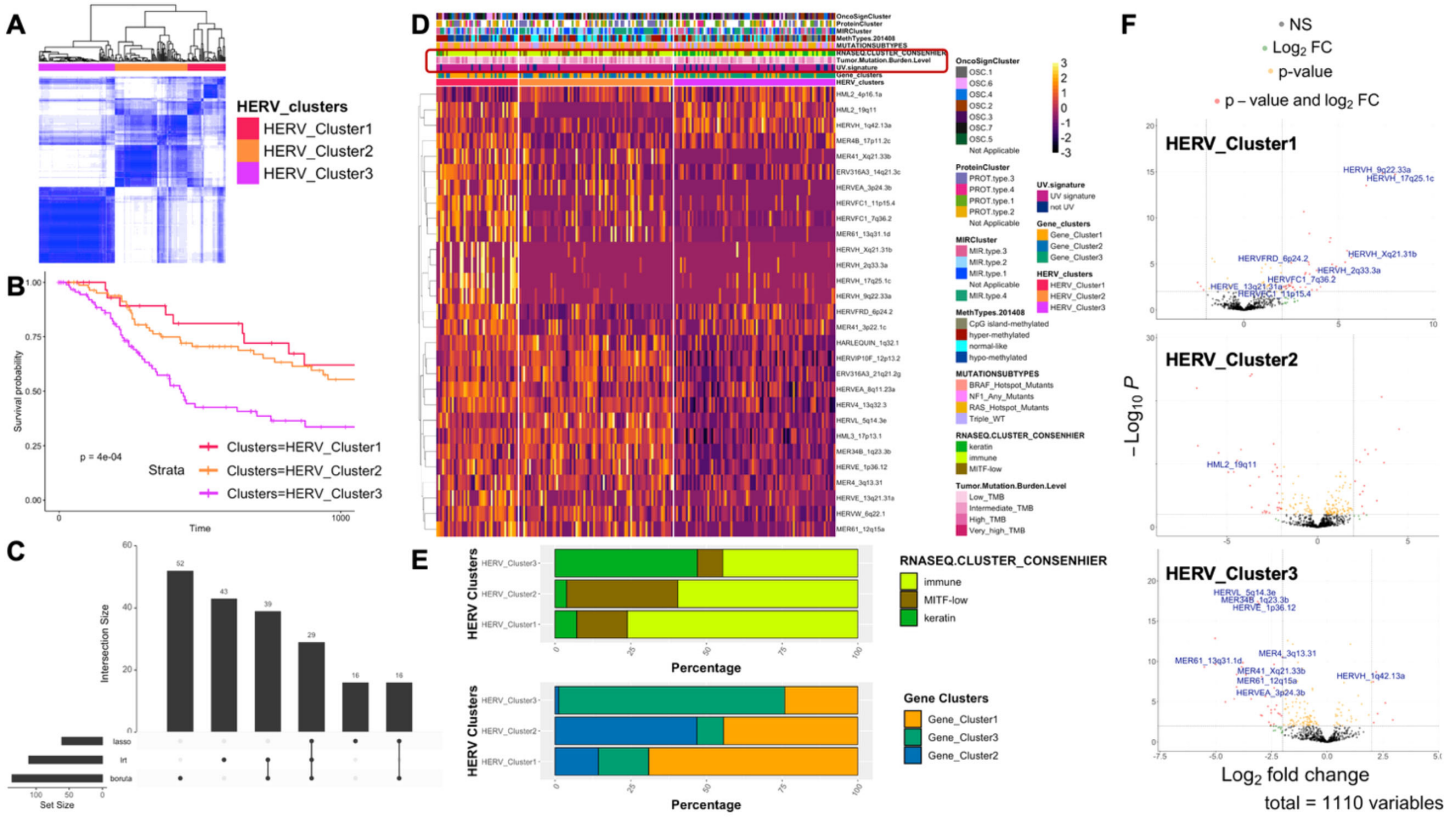
Metastatic cutaneous melanoma samples separate into 3 clusters based on HERV expression and is related to survival outcome. **(A)** Unsupervised clustering of metastatic melanoma samples based on HERV expression, using k-means algorithms with Euclidean distance. **(B)** Kaplan-Meier survival analysis of the 3 clusters for metastatic melanoma calculated by gene expression. Showing the first 1000 days of survival probability. The clusters are renamed based on overall survival probability: “3” = “HERV_Cluster1”, “2” = “HERV_Cluster2”, and “1” = “HERV_Cluster3”. P-value for the survival clusters is shown on the graph. **(C)** Upset plot showing the number of differential HERVs distinguishing the clusters calculated by each feature selection methods. **(D)** Stack plot showing the percentage composition of each cluster classified from the original literature and gene expression calculated clusters in the clusters calculated from HERV expression. Annotation for clustering results, tumor mutation burden level and UV signature are highlighted. **(E)** Heatmap showing expression of HERVs selected by all feature selection methods (LRT, BORUTA, LASSO) in each sample. Rows are labeled by the HERV names and columns are annotated by the HERV cluster, gene cluster, UV signature, clusters calculated by RNA signature, mutation subtypes, methylation types, miRNA cluster, protein cluster, and oncogene signature cluster in original literature. **(F)** HERV volcano plot for each cluster vs. all other clusters, from top to down: HERV_Cluster1 vs. other, HERV_Cluster2 vs. other, and HERV_Cluster3 vs. other. The HERVs that are selected by all 3 feature selection methods are annotated for their loci names.

We filtered from the 265 metastatic samples for complete metadata on sample sites and survival outcome. From the 204 filtered samples (**Supplementary Figure 1**), we performed unsupervised clustering based on gene and HERV expression independently (**Figure 2A**; **Supplementary Figure 3A**). Three clusters defined by gene and HERV expression showed a significantly different survival outcome (*p* = 4e^-4^). These cluster groups were renamed based on the survival probability (**Figure 2B**; **Supplementary Figure 3B**).

For differential gene analysis, the top feature-selected genes were classified to belong to the immune response functional group, and for the genes selected by feature selection methods, most were downregulated in gene cluster 3, the worst-prognosis cluster (**Supplementary Figures 3C, D, S4I**). For the HERV cluster group, 29 differentially expressed HERVs were selected by three feature selection methods from a variety of HERV clades and families. Most of the consensus HERVs were down-expressed in the worst-survival HERV cluster 3 (**Figures 2C, D**). When cross- compared with the described “immune”, “keratin”, and “MITF-low” clusters in the original literature (16), the gene clusters showed a high association with an adjusted Rand index of 0.322, while the HERV clusters showed some associations, but not as similar as the genes (adjusted Rand index = 0.065) (**Figure 2E**; **Supplementary Figures 3D, E; S4A, B, E, F**, **Supplementary Table 2**). This was expected as gene RNA expression was one of the factors considered when the original clustering was calculated, but not HERV expression.

Both HERVs and gene clusters were associated with the UV signature of the patients, where the group with “not UV” signature fell mostly into the groups possessing poorer prognostic outcomes (**Supplementary Figures 4C, G**). However, for tumor mutation burden level, we only found a weak association with gene and HERV clusters (Rand index for gene clusters = 0.012 and HERVs = 0.001) (**Supplementary Figures S3F**; **S4D, H**, **Supplementary Table 2**). HERV expression was down regulated in the group with the poorest survival outcome, but several HERVs such as HERVH 1q42.13a and ERVB4 1q32.2b still showed significant upregulation in HERV cluster 3 (**Figure 2F**).

Although none of the seven differentially expressed HERVs distinguishing primary and metastatic groups were reported to characterize the metastatic subtypes shown here, we observed similar groups of HERVs such as HERVH, MER61 (MER61_12q15a), and HERVK/HML2 (HML2_4p16.1a, HML2_19q11) to be significantly upregulated or downregulated in the worst prognosis cohort (**Figures 1F**, **2D**). In addition, MER61_12p13.31b and HERVFRD_6p24.2 were found to be differentially expressed (selected by at least one feature selection method) in both analyses. These results confirm that certain dysregulated HERVs were associated with poor prognosis and melanoma molecular subtypes.

### 3.3 HERV expression level and melanoma immunotherapy outcome

Next, we focused on patients with metastasized melanomas who were treated with ICI and whose survival outcome had been reported. To do this, we incorporated another dataset containing an additional 27 RNA-seq CM samples (29). Initially, we planned to combine the 20 samples in TCGA-SKCM with recorded treatment outcome with the anti-PD1 batch. However, the evaluation categories for the TCGA cohort varied from the anti-PD1 study, which adopted the commonly recognized RECIST evaluation criteria (33). This discrepancy, combined with the data collection protocol differences (16, 29), resulted in failed RNA-seq batch corrections (**Supplementary Figures 5A, B**), whereas, in contrast, the batch correction method performed well without incorporating the TCGA cohort (**Supplementary Figures 5E, F**). We thus decided to perform the analysis comparing immunotherapy response independently for the anti-PD1 and TCGA datasets.

**Figure 3:**
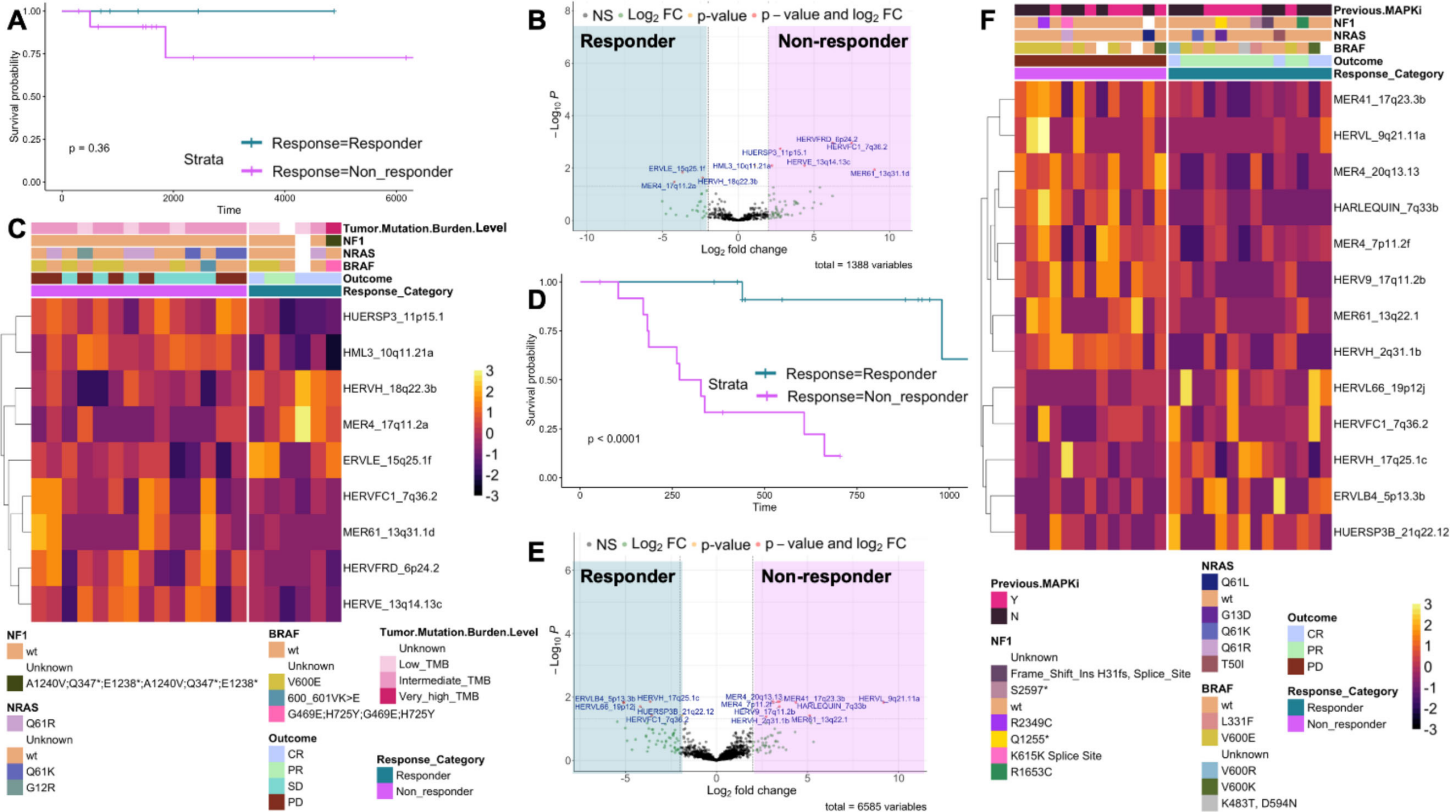
Up-regulated HERV expression in pre-treatment samples of non-responder to immunotherapy patients. **(A)** Kaplan-Meier survival analysis of the response outcome groups in the TCGA dataset. Complete response (CR) and partial response (PR) are classified as “Responder”; Stable disease (SD) and Progressive disease are classified as “Non-responder). P-value for the survival clusters is shown on the graph. **(B)** HERV volcano plot for responders vs. non-responders in the TCGA dataset. The HERVs that are significant (absolute Log2FC > 2 and P < 0.05) are annotated for their loci names. **(C)** Heatmap showing expression of significant DE HERVs between the outcome groups in the TCGA analysis for each sample. Rows are labelled by the HERV locus and columns are annotated by response, outcome, BRAF mutation type, NRAS mutation type, NF1 mutation type, and the tumor mutation burden level. **(D)** Kaplan-Meier survival analysis of the response outcome groups in the RECIST dataset. Complete response (CR) and partial response (PR) are classified as “Responder”; Progressive disease is classified as “Non-responder). P-value for the survival clusters is shown on the graph. **(E)** HERV volcano plot for responders vs. non-responders in the RECIST dataset after batch correction. The HERVs that are significant (absolute Log2FC > 2 and P < 0.05) are annotated for their loci names. **(F)** Heatmap showing expression of significant DE HERVs between the outcome groups after batch correction in the RECIST analysis for each sample. Rows are labelled by the HERV locus and columns are annotated by response, outcome, BRAF mutation type, NRAS mutation type, NF1 mutation type, and if the patients have received previous MAPKi treatment.

For the TCGA dataset, we conducted a KM survival analysis comparing the two outcome groups. We observed a difference in survival; however, it was not statistically significant (*p* = 0.36), which was likely due to the small sample size (responders *n* = 6, non-responders *n* = 14) (**Figure 3A**; **Supplementary Table 1C**). When plotting samples based on HERV expression using PCA, we saw that the response groups separated in the PC1 and PC2 space (**Supplementary Figure 5C**). Furthermore, both immunotherapy outcome and TMB level were associated, with a Rand index of 0.412 (**Supplementary Figure S5D**), agreeing with previous literature (32). By performing differential expression analysis, we found that a higher number of HERVs were upregulated, and similar patterns for differentially expressed genes were observed (**Figures 3B, C**; **Supplementary Figures 6A, B**). No obvious association between gene mutation types and immunotherapy outcome were found (**Figure 3C**).

When investigating the RECIST dataset, which contained similar numbers of non-responders and responders, patient survival outcome was significantly correlated with the treatment response (*p* < 0.0001). The responders were predicted to have higher than 75% survival probability, but the non-responder group was predicted to have lower than 25% survival probability in a 750-day time span (**Figure 3D**).

We found a similar trend of non-responders having a higher number of HERVs and genes upregulated in non-responders (**Figures 3E, F**; **Supplementary Figures  6E, F**). A total of eight downregulated HERVs and 14 upregulated HERVs were found (**Figures 3B, C, E, F**) and plotted to confirm the different count distribution for each outcome group (**Supplementary Figures 5G,H**). However, we found that HERVFC1_7q36.2 was downregulated in the non-responder group in the TCGA dataset but upregulated in the non-responder group in the RECIST dataset (**Supplementary Figures 5G, H**). We suspect that this might be a false positive candidate or this inconsistency might be due to a different environmental or therapeutic exposure associated with the separate cohorts. We thus did not consider this HERV as relevant to the specific anti-PD1 response of interest and excluded it from downstream analyses. We continued to observe that HERVFRD_6p24.2 and MER61_13q31.1d were significantly upregulated in non-responder groups (**Figures 3B,E**). To conclude, we observed many dysregulated HERVs in the metastatic compared to the primary group.

### 3.4 Upregulated HERVs

**Figure 4:**
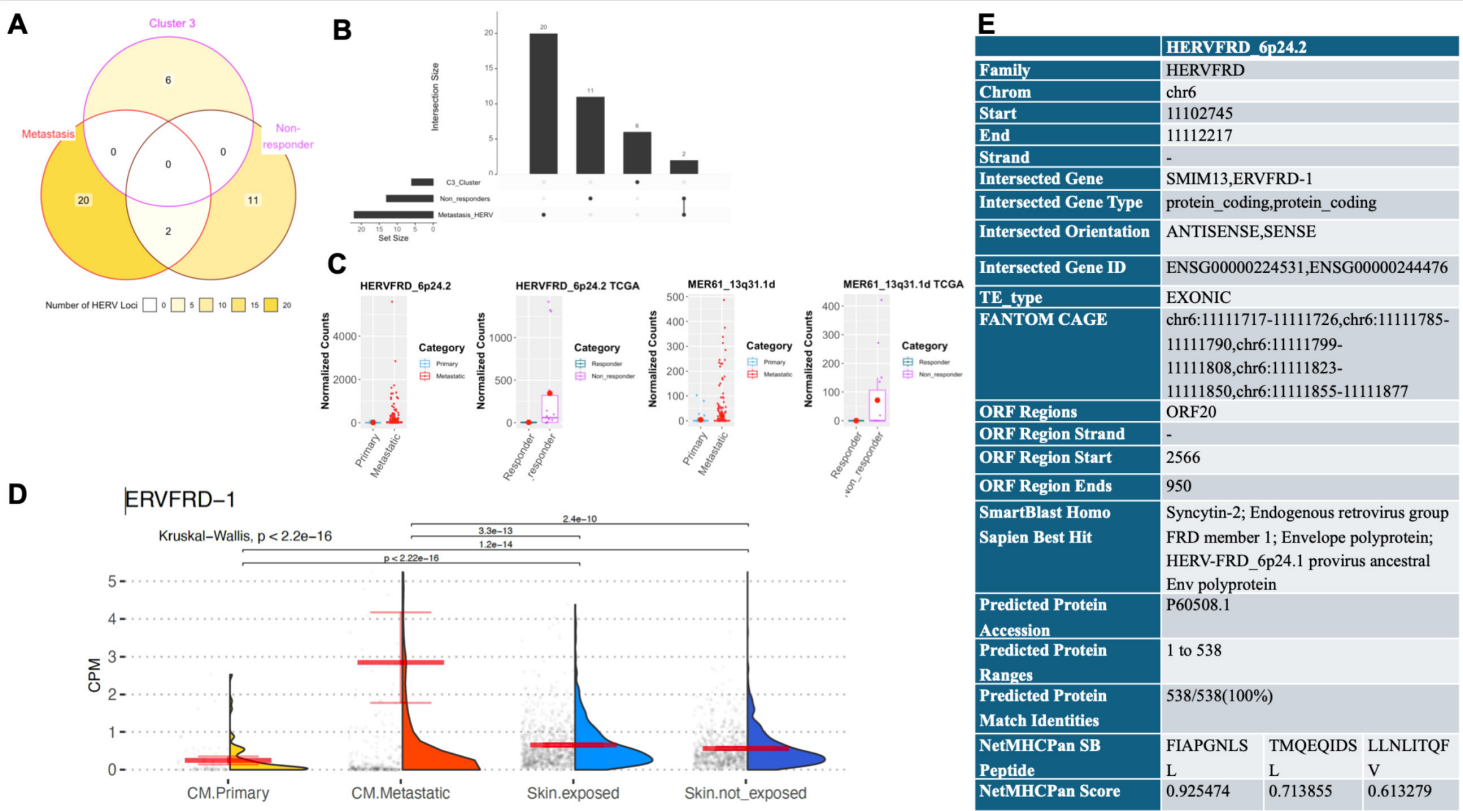
HERVs dysregulated in metastasis, the worst prognostic cluster and non-responder groups are distinct and have translation potential. **(A)** Venn Diagram showing the overlapping and different HERVs significantly upregulated in metastatic melanoma, C3 worst diagnostic metastatic samples, and patients who are non- responders after immunotherapy treatment. For the progressive disease group, HERVs are selected from **Supplementary Figure S5H** but excluded HERVFC1_7q36.2 because it is upregulated in the TCGA dataset but downregulated in the Hugo dataset. **(B)** Upset plot showing the HERVs upregulated in metastatic melanoma, C3 worst diagnostic metastatic samples, and patients developing progressive disease after immunotherapy treatment. **(C)** Normalized count plots showing the consensus HERVs upregulated in metastatic vs. primary and non-responders vs. responders. **(D)** Expression of ERVFRD-1 in cutaneous melanoma and normal skin tissues from the Genotype Tissue Expression (GTEx) consortium. Read counts for skin samples were obtained from GTEx v11 data. Counts were normalized by library size to obtain counts per million (CPM). For each group, the mean and 95% confidence limits calculated by non-parametric bootstrap are shown in red. Among all groups, the means are significantly different (Kruskal-wallis, p < 2.2e-16). Significant pairwise comparisons (wilcoxon rank sum test, p.adj < 1e-3) are shown above the plots.CPM scale on y-axis is zoomed into y-limit >=0 and <=6 for better visualization. **(E)** Summary of Epitope search results for HERVFRD_6p24.2.

Two consensus HERVs were upregulated in the metastatic subgroup compared to the primary CM group and in the immunotherapy non-responders (**Figures 4A–C**; **Supplementary Figures 7A, B**). We looked for potential protein coding regions from HERVs HERVFRD_6p24.2 and MER61_13q31.1d by using NCBI ORFfinder with the hg38 (35) annotation. Since HERVFRD_6p24.2 overlaps with the canonical gene ERVFRD-1, we asked whether the expression of ERVFRD-1 was unique to primary CM and metastases or whether it is expressed in physiologic conditions. Using gene expression data from 19,788 normal tissue samples (GTEx consortium) we examined the expression of ERVFRD-1 in normal tissues and compared with CM tumors (**Supplementary Figures 8**). Of all tissues, metastatic CM has the greatest average expression, though the distribution appears skewed by many samples with extreme CPM values (**Supplementary Figures 8B**). However, ERVFRD-1 does not have tumor-specific expression, and is highly expressed in other tissues, including Bladder, Small Intestine, and Esophagus (mean CPM=1.72,1.44, and 1.40, respectively). Since primary CM originates from cutaneous melanocytes, we were particularly interested in whether ERVFRD-1 is expressed in normal skin. We found that ERVFRD-1 expression is significantly higher in CM compared with both sun-exposed skin and non-exposed skin (**Figure 4D**).

We used blastp on the swissprot database (38) and predicted proteins were evaluated with NetMHCPan (39) for potential binding to a common HLA molecule. We identified ORFs within the MER61_13q31.1d transcript (**Supplementary Table 3**). The ERVFRD_6p24.2 ORF20 was predicted to code for syncytin-2, a known Env in the ERV-FRD group. Three peptides from this protein region were predicted to bind strongly bind to the HLA-A02:01 allele (**Figure 4E**). These 3 peptides are within the TM domain of ERVFRD *Env* but are not in the immunosuppressive domain region (45).

## 4 Discussion

In this study, we compared the HERV expression profiles between metastatic and primary CM and performed subtype categorization of CM using HERV expression. We used the Telescope (34) pipeline to quantify HERV counts from bulk RNA-seq datasets. We saw a pattern of HERV upregulation in ICI non-responders when compared to responders and identified a HERV-FRD locus upregulated in metastatic CM and in ICI non-responders. This HERV-FRD locus encodes a syncytin-2 protein. We saw that HERV expressions were significantly related to CM subtypes and highlight the potential in the future of assessing HERV activity in CM.

In the context of tumor progression, the upregulation of genes and HERVs is frequently reported (10, 22, 40, 46–48). However, we observed HERV downregulation in CM metastases. We predict that (1) malignant metastatic cells might have developed some immune escape mechanisms to suppress checkpoint inhibitors and directly or indirectly suppressed HERVs in proximate regions or (2) discrepancies in metastasized vs. primary CM cell-type abundances contributed to differential HERV activity.

HERV products form recurrent cancer-specific neoantigens and are promising targets for immunotherapy (18–20, 24, 40–42, 48–56). In this study, we found a HERV-FRD locus which has the potential to produce syncytin-2 protein. Although many HERV loci exhibit high similarity in their sequences, it has been reported that they vary largely in translation potentials, synthesized peptides and protein functions (57, 58). Here syncytin-1 and syncytin-2 are HERV-W and HERV-FRD *Env*, respectively, that are expressed in the human placenta where they facilitate cell-to-cell fusion and syncytiotrophoblast formation (59). While these proteins possess crucial roles for human reproduction, deregulated HERV activity can be associated with somatic toxicity — for example, HERV-W is overexpressed in cutaneous T-cell lymphoma (CTCL), some hematological malignancies, colorectal cancer, gynecological cancer, urological cancer, and endocrine cancer (40). Here we speculate that overexpression of syncytin-2 might produce protein products that could be processed and presented by MHC alleles and could therefore potentially be an immunotherapeutic target. Syncytin-2 has also been found to be immunosuppressive, and its ectodomains can potentially trigger antibody responses (60). Future works building upon this finding would need to confirm the translation of syncytin-2 products and validate the MHC presentation of syncytin-2 processed peptides before developing and testing syncytin- 2-targeting strategies.

Our study possesses certain limitations, and thus we caution about over-interpretation of these early findings. First, the small sample size of the responder treatment outcome group (*n* = 6 in the TCGA) results in a low capture rate of significantly differential HERVs. Future studies that would increase the sample size, in tandem with similar outcome evaluation categories, patient demographics, and library preparation workflow, would help to identify additional deregulated HERV elements. Second, future work is needed to confirm the exact coding potential, antigenic properties, physiological importance, position, and sequence of CM-specific syncytin-2. Third, this study was performed entirely using retrospective cohorts, and access to samples for biological validation or follow-up studies was limited. Fourth, the cohorts utilized within this study demonstrate substantial batch effects that hindered integration and translation as previously indicated. Larger cohorts that consider temporal changes in HERV expression with follow-up consultations and biopsies could be implemented to address these limitations. Fifth, this study utilized short-read bulk RNA sequencing for HERV expression quantification and thus discards the potential impact of HERV isoforms and cell- type-specific expression profiling. Future work that utilizes long-read RNA sequencing together with single-cell approaches could better define HERV isoforms and uncover the cell types responsible for differential HERV expression as well as describe the novel HERV expression profiles of occult cell types in the tumor microenvironment.

This study provides a novel investigation into HERV expression dynamics in the metastasis and subtyping of CM. We found that HERVs were generally downregulated in metastasized samples collected from patients with melanoma. However, within the group of ICI non-responders, certain HERVs were significantly upregulated. We identified syncytin-2 as a putative biomarker of CM pathophysiology and suggest that further investigation is warranted. In summary, our study demonstrates an emerging role for HERV expression in the tumor immune microenvironment and CM metastasis.

## Data Availability

Existing datasets are available in a publicly accessible repository: Publicly available datasets were analyzed in this study. This data can be found here: https://www.cancer.gov/ccg/research/genome-sequencing/tcga/studied-cancers/cutaneous-melanoma-study and here: https://pubmed.ncbi.nlm.nih.gov/26997480/.
