# Supplementary material for: Human endogenous retrovirus profiling reveals heterogenous expression in cutaneous melanoma
Source: Front Oncol. 2026 Mar 26;16:1708501. doi: 10.3389/fonc.2026.1708501 (PMC13061668; doi:10.3389/fonc.2026.1708501)
Supplement: Supplementary file 1 [file Supplementaryfile1.pdf]

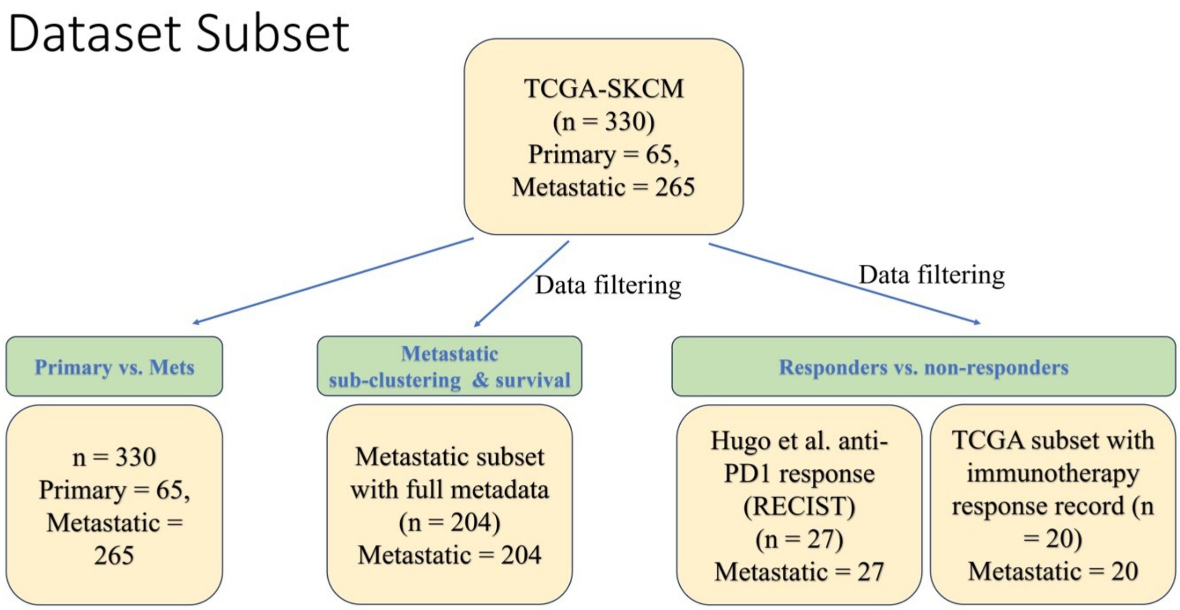

Fig.S1: Data retrieval and subset

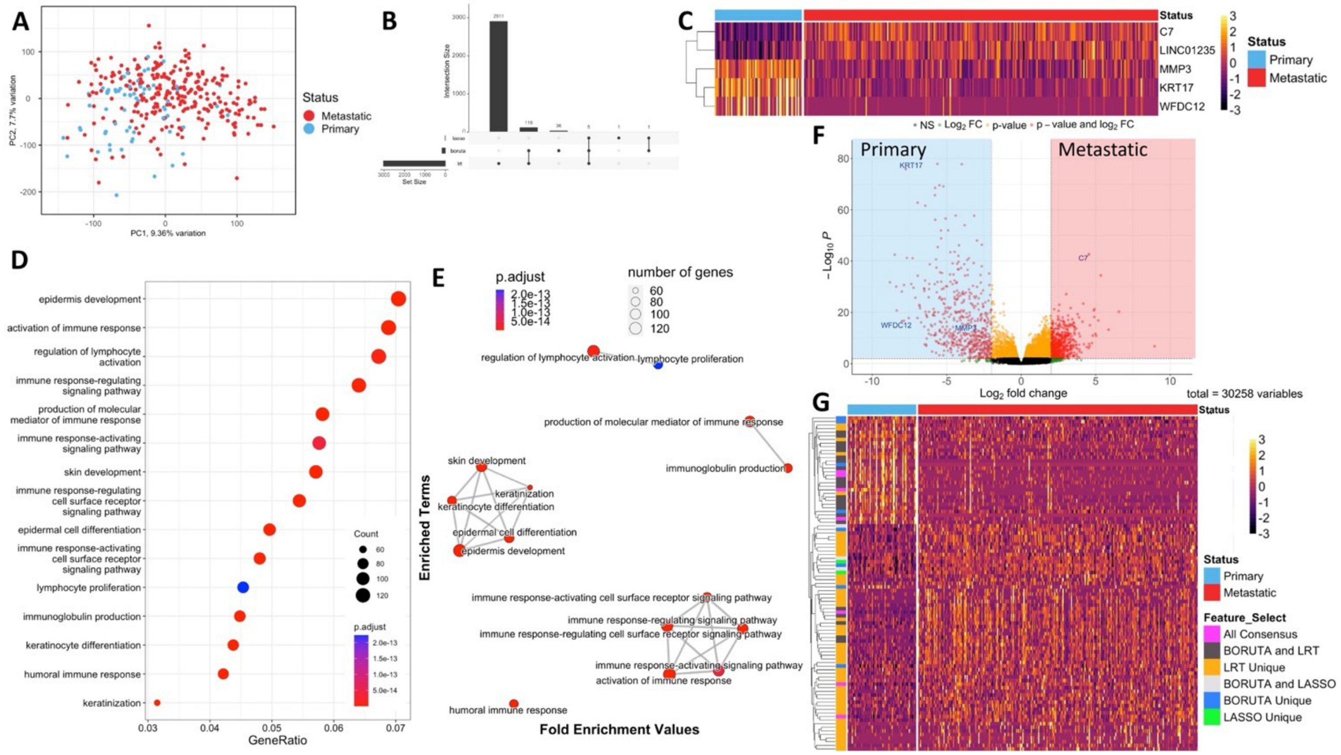

Fig.S2: Gene and HERV differential analysis between primary and metastatic melanoma

S2A: PCA of gene expression in all TCGA-SKCM samples. Genes representing the lowest 30% of variation are removed. Colored based on the sample status (metastatic or primary tumor).

S2B: Upset plot showing the number of differential genes calculated by each feature selection methods.

S2C: Heatmap showing expression of 5 consensus feature selected genes in each sample.

S2D: Using all genes selected by any feature selection methods (FS1B), enrichment analysis is performed, and top 15 categories are shown. Dot size represents the number of genes in each marked function category, and dot color represents the adjusted p value of each function category.

S2E: Using all genes selected by any feature selection methods (FS1B, FS1D), enrichment analysis is performed, and enrichment map is visualized for the top 20 functional categories. Dot

size represents the number of genes in each marked function, and dot color represents the adjusted p value of each function. Lines are connecting functions into each systematic group.

S2F: Volcano plot of differential gene analysis between primary and metastatic melanoma samples. P values and log fold change are calculated with the Wald model. Marked genes are consensus feature selected genes using LRT, BORUTA and LASSO methods. P-values cutoff = 0.01, and Log2FC cutoff = +/- 2.

S2G: Heatmap showing expression of HERVs selected by any of the feature selection methods (LRT, BORUTA, LASSO) in each sample. Rows are annotated by the which method each gene is selected from and columns are annotated by the status of the sample (primary of metastatic).

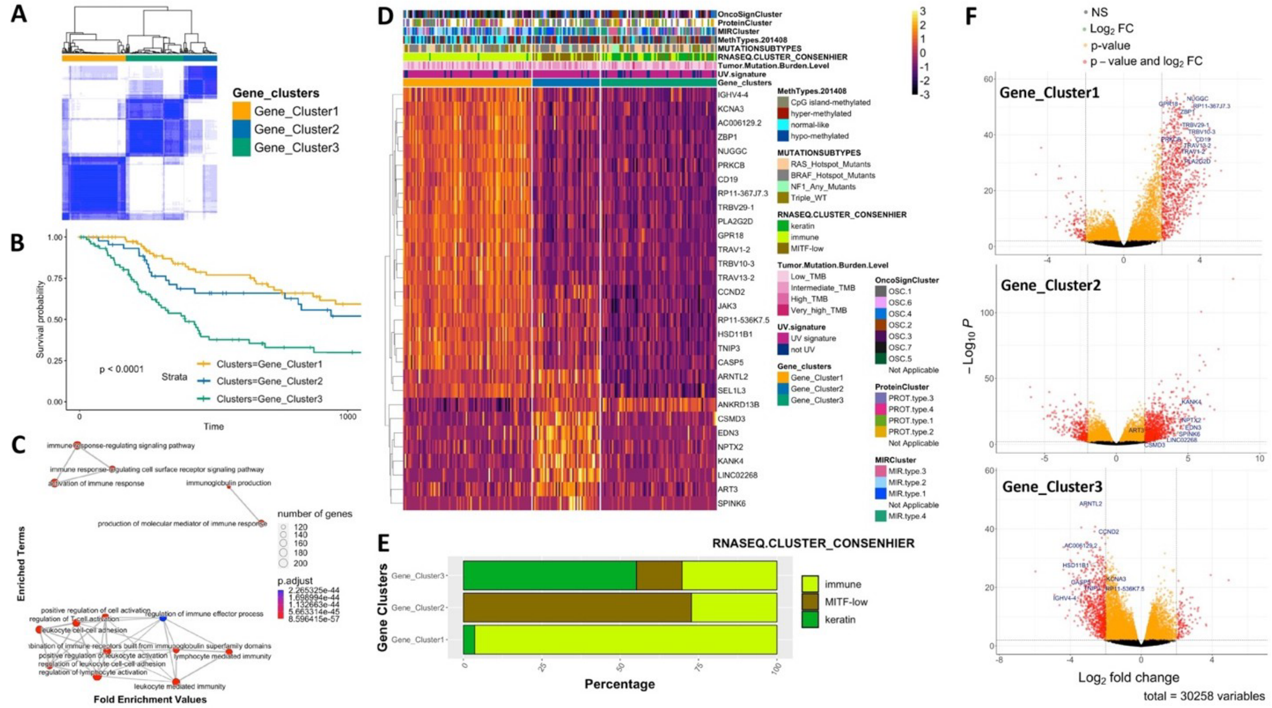

Fig.S3: Metastatic cutaneous melanoma samples separate into 3 clusters based on gene expression and is related to survival outcome

S3A: Unsupervised clustering of metastatic melanoma samples based on gene expression, using k-means algorithms with Euclidean distance.

S3B: Kaplan-Meier survival analysis of the 3 clusters for metastatic melanoma calculated by gene expression. Showing the first 1000 days of survival probability. The clusters are renamed based on overall survival probability: “3” = “Gene Cluster 2”, “2” = “Gene Cluster 1”, and “1” = “Gene Cluster 3”. P-value for the survival clusters is shown on the graph.

S3C: Using all genes selected by any feature selection methods to distinguish the clusters, enrichment analysis is performed, and enrichment map is visualized for the top 15 functional categories. Dot size represents the number of genes in each marked function, and dot color represents the adjusted p value of each function. Lines are connecting functions into each systematic group.

S3D: Stack plot showing the percentage composition of each cluster classified from the original literature in the clusters calculated from gene expression.

S3E: Heatmap showing expression of genes selected by all feature selection methods (LRT, BORUTA, LASSO) in each sample. Rows are labelled by the gene names and columns are annotated by the gene cluster, UV signature, clusters calculated by RNA signature, mutation subtypes, methylation types, miRNA cluster, protein cluster, and oncogene signature cluster in original literature.

S3F: Gene volcano plot for each cluster vs. all other clusters, from top to down: Gene_Cluster1 vs. other, Gene_Cluster2 vs. other, and Gene_Cluster3 vs. other. The genes that are selected by all 3 feature selection methods are annotated for their gene names.

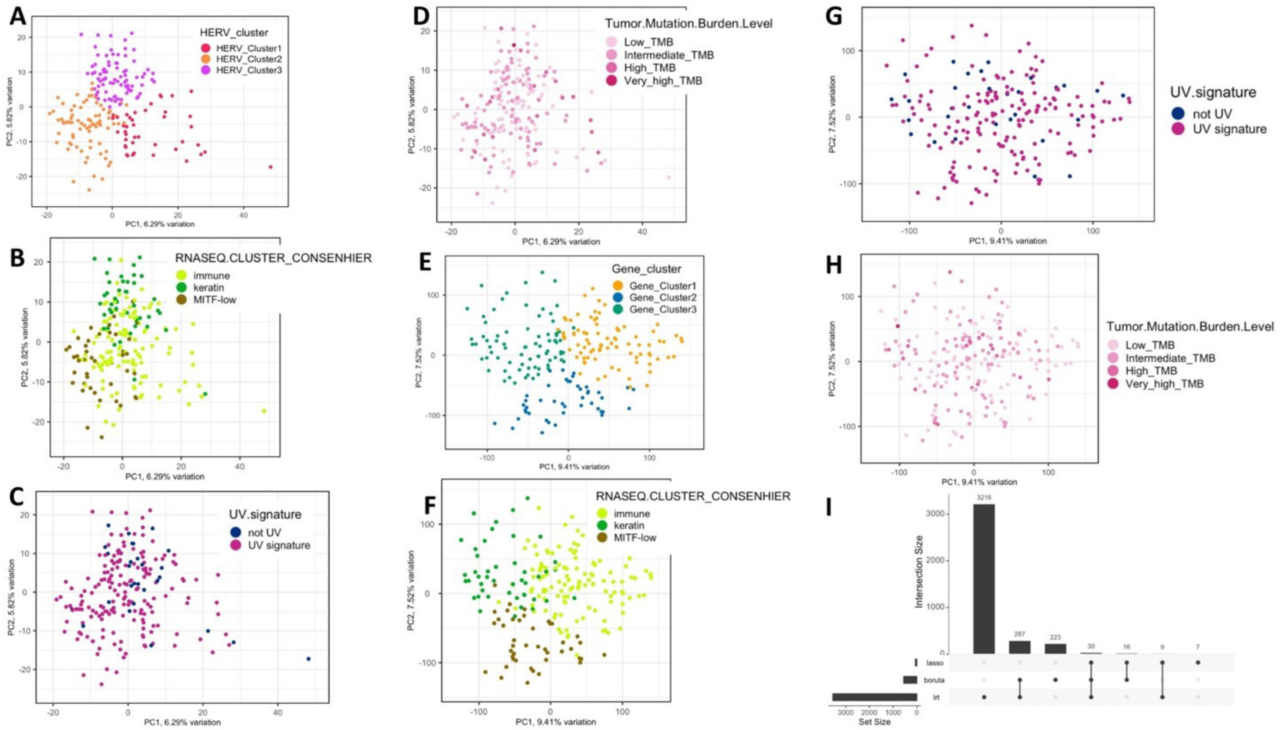

Fig.S4: Gene and HERV expression clusters in metastatic melanoma is consistent as previously described and are associated with UV signature

S4A: PCA plot of metastatic melanoma HERV expression, colored by the unsupervised HERV clusters.

S4B: PCA plot of metastatic melanoma HERV expression, colored by the computed clusters by RNA sequencing in the original publication.

S4C: PCA plot of metastatic melanoma HERV expression, colored by UV signature.

S4D: PCA plot of metastatic melanoma HERV expression, colored by tumor mutation burden level.

S4E: PCA plot of metastatic melanoma gene expression, colored by the unsupervised gene clusters.

S4F: PCA plot of metastatic melanoma gene expression, colored by the computed clusters by RNA sequencing in the original publication.

S4G: PCA plot of metastatic melanoma gene expression, colored by UV signature.

S4H: PCA plot of metastatic melanoma gene expression, colored by tumor mutation burden level.

S4I: Upset plot showing the number of differential genes distinguishing the clusters calculated by each feature selection methods.

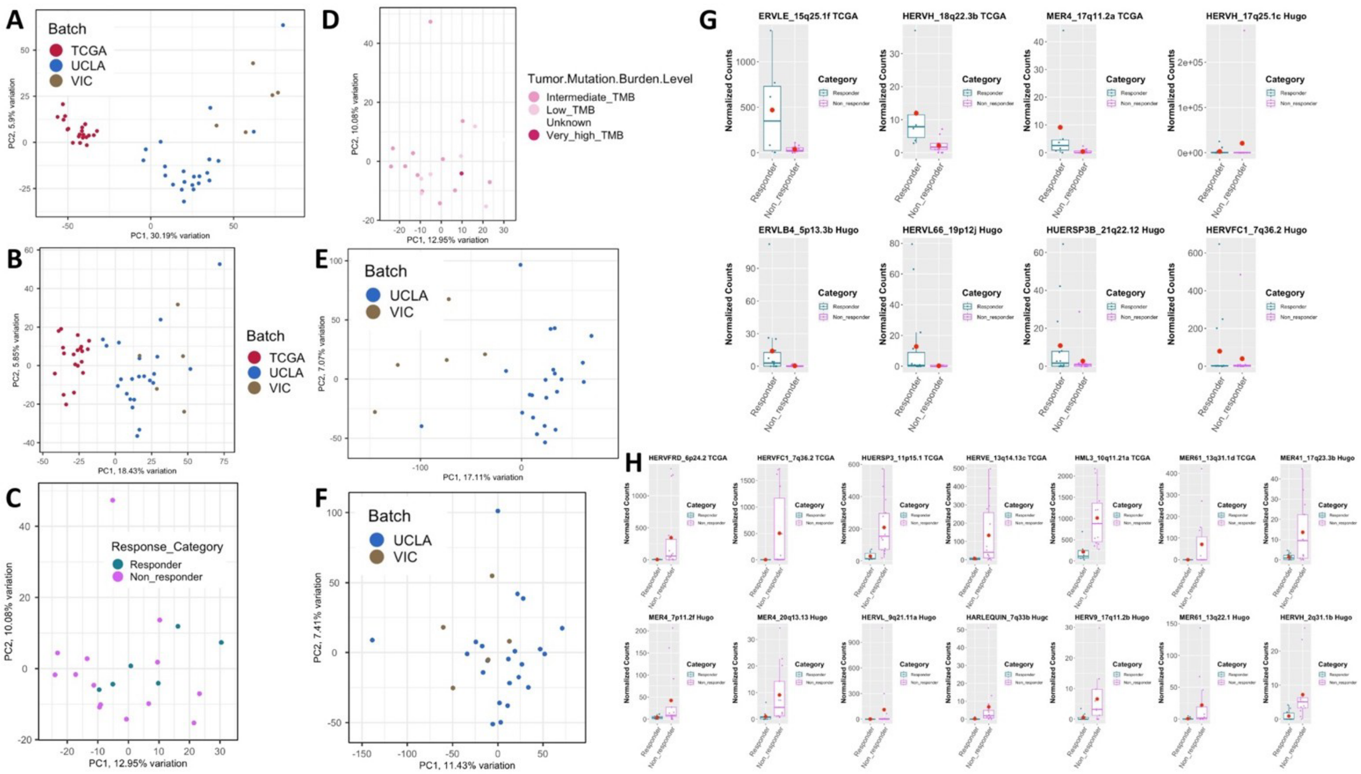

Fig.S5: Anti-PD1 and TCGA datasets cannot be combined for the response outcome analysis.

S5A: PCA plot of HERV expression in the anti-PD1 treatment and TCGA datasets combined, colored by batches (TCGA, UCLA or Vanderbilt-Ingram Cancer Center).

S5B: PCA plot of HERV expression in the anti-PD1 treatment and TCGA datasets combined after corrected by combat-seq, colored by batches.

S5C: PCA plot of HERV expression in the TCGA treatment subset, colored by response category.

S5D: PCA plot of HERV expression in the TCGA treatment subset, colored by tumor mutation burden level.

S5E: PCA plot of HERV expression in the anti-PD1 treatment dataset, colored by batches.

S5F: PCA plot of HERV expression in the anti-PD1 treatment dataset after corrected by combat-seq, colored by batches.

S5G: Normalized count plots showing HERVs down-regulated in non-responders vs. responders for the 2 datasets. Top panel is from the TCGA dataset and bottom panel is from the anti-PD1

dataset. The line indicates median and red dot indicates mean normalized count of each sub-group.

S5H: Normalized count plots showing HERVs upregulated in non-responders vs. responders for the 2 datasets. Top panel is from the TCGA dataset and bottom panel is from the anti-PD1 dataset. The line indicates median and red dot indicates mean normalized count of each sub-group.

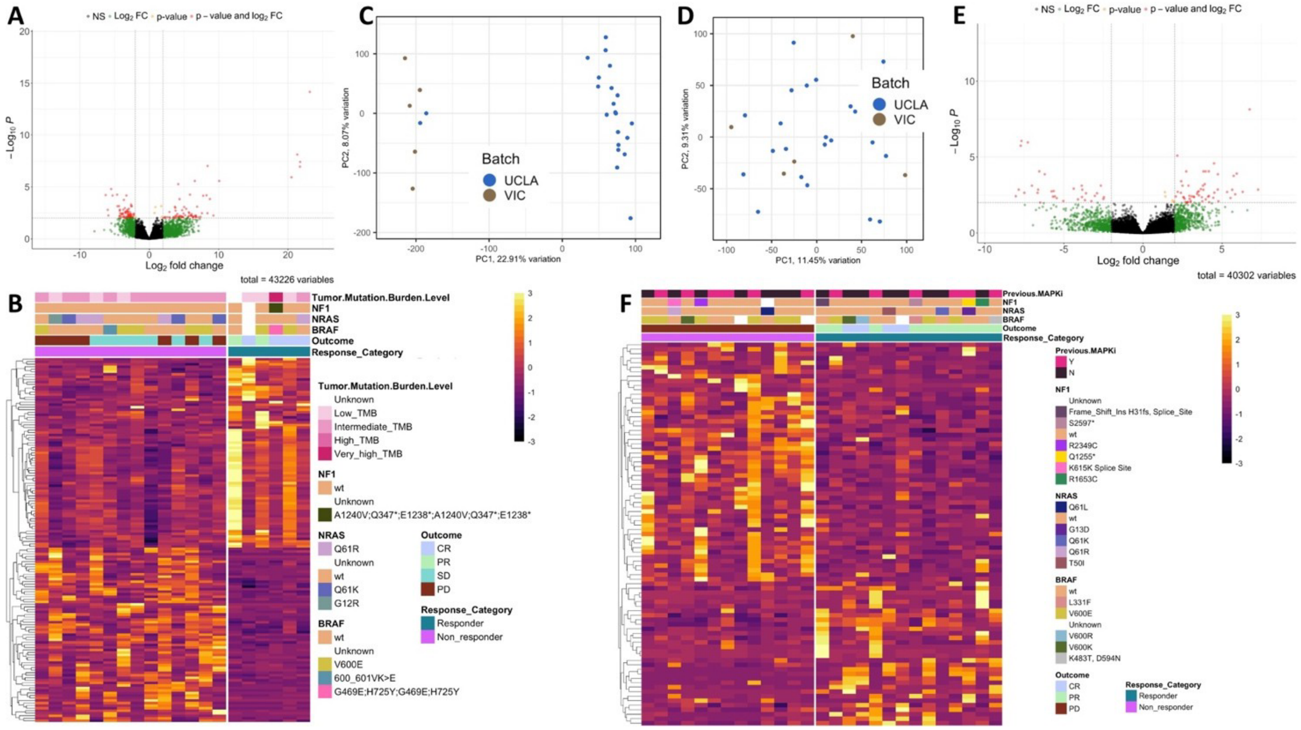

Fig.S6: Genes expression is dysregulated in samples from non-responder patients.

S6A: Volcano plot showing the non-responder vs. responder DE gene analysis in TCGA dataset . The threshold for significant HERVs are set to absolute Log2FC > 1 and P < 0.05.

S6B: Heatmap showing expression of significant DE genes between the outcome groups in the TCGA analysis for each sample. Rows are labelled by the HERV locus and columns are annotated by response, outcome, BRAF mutation type, NRAS mutation type, NF1 mutation type, and the tumor mutation burden level.

S6C: PCA plot of gene expression in the anti-PD1 treatment dataset, colored by batches (UCLA or Vanderbilt-Ingram Cancer Center).

S6D: PCA plot of gene expression in the anti-PD1 treatment dataset after corrected by combat-seq, colored by batches.

S6C: PCA plot of gene expression in the anti-PD1 treatment dataset after correction by combat-seq, colored by the outcome of treatment.

S6E: Volcano plot showing the non-responder vs. responder DE gene analysis in anti-PD1 dataset corrected by batches. The threshold for significant HERVs are set to absolute Log2FC > 1 and $P < 0.05$.

S6F: Heatmap showing expression of significant DE genes between the outcome groups in the anti-PD1 dataset corrected by batches for each sample. Rows are labelled by the HERV locus and columns are annotated by response, outcome, BRAF mutation type, NRAS mutation type, NF1 mutation type, and if the patients have received previous MAPKi treatment.

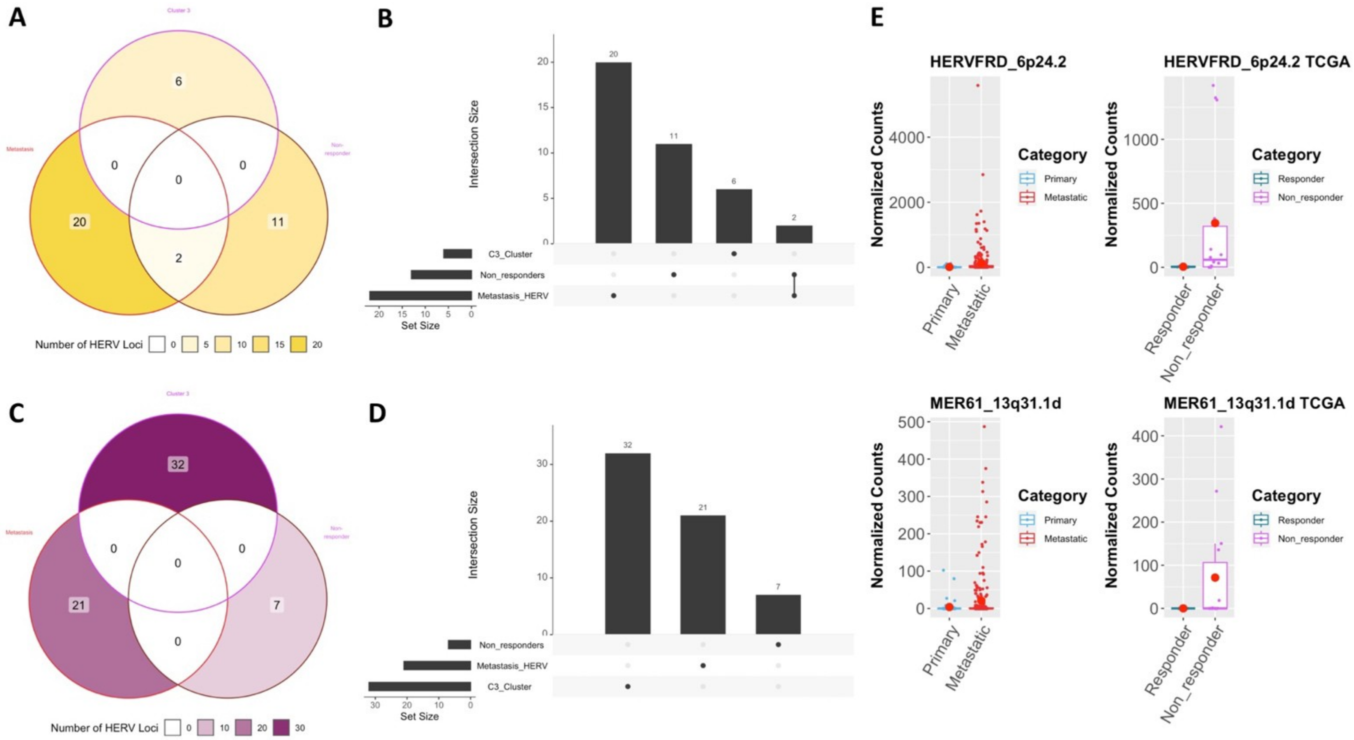

Fig.S7: HERVs dysregulated in metastasis, worst prognostic cluster and non-responder groups are distinct

S7A: Venn Diagram showing the overlapping and different HERVs significantly upregulated in metastatic melanoma, C3 worst diagnostic metastatic samples, and patients who are non-responders after immunotherapy treatment. For the progressive disease group, HERVs are selected from Figure S5H but excluded HERVFC1_7q36.2 because it is upregulated in the TCGA dataset but downregulated in the Hugo dataset.

S7B: Upset plot showing the HERVs upregulated in metastatic melanoma, C3 worst diagnostic metastatic samples, and patients developing progressive disease after immunotherapy treatment.

S7C: Venn Diagram showing the overlapping and different HERVs significantly downregulated in metastatic melanoma, C3 worst diagnostic metastatic samples, patients who are non-responders after immunotherapy treatment. For the progressive disease group, HERVs are

selected from Figure S5G but excluded HERVFC1_7q36.2 because it is upregulated in the TCGA dataset but downregulated in the Hugo dataset.

S7D: Upset plot showing the HERVs downregulated in metastatic melanoma, C3 worst diagnostic metastatic samples, and patients developing progressive disease after immunotherapy treatment.

S7E: Normalized count plots showing the consensus HERVs upregulated in metastatic vs. primary and non-responders vs. responders.

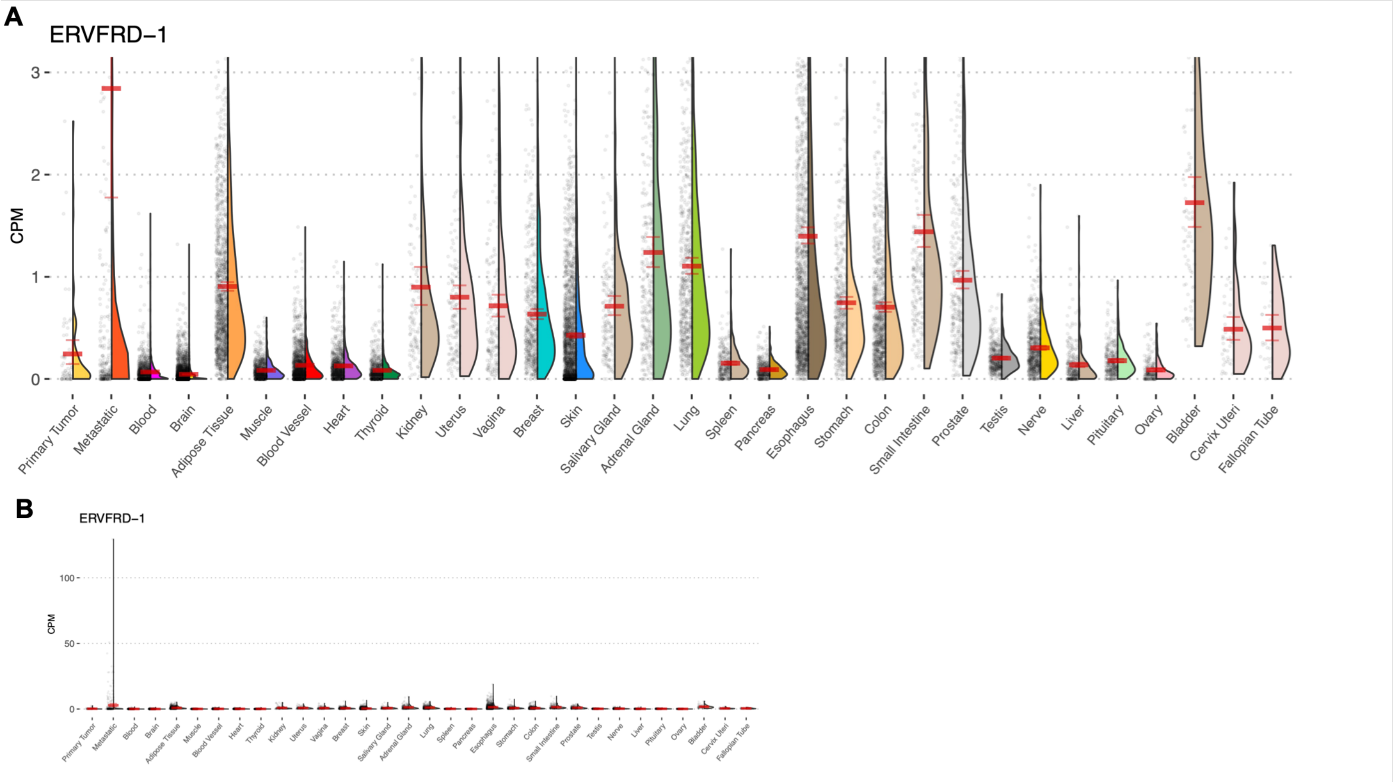

Fig. S8. Expression of ERVFRD-1 in cutaneous melanoma and normal tissues. Expression of ERVFRD-1 in cutaneous melanoma (this study) is compared with normal tissues from the Genotype Tissue Expression (GTEx) consortium. Read counts were obtained as previously described for this study and from GTEx v11 data. Counts were normalized by library size to obtain counts per million (CPM). (A) The expression of ERVFRD-1 in primary CM, metastatic CM, and GTEx tissue samples, grouped by tissue. For each group, the mean and 95% confidence limits calculated by non-parametric bootstrap are shown in red. Plot is truncated at CPM=3.0 for visibility. (B) The expression of ERVFRD-1 grouped by tissue, as in A, without truncation.
